# Clinical significance of soluble immunoglobulins A and G and their coated bacteria in feces of patients with inflammatory bowel disease

**DOI:** 10.1186/s12967-018-1723-0

**Published:** 2018-12-17

**Authors:** Ritian Lin, Hongwei Chen, Weigang Shu, Mingming Sun, Leilei Fang, Yanhong Shi, Zhi Pang, Wei Wu, Zhanju Liu

**Affiliations:** 1 Department of Gastroenterology, The Shanghai Tenth People’s Hospital of Tongji University, No. 301 Yanchang Road, Shanghai, 200072 China; 2 Department of Gastroenterology, Luoyang Central Hospital Affiliated to Zhengzhou University, No. 288 Middle Zhongzhou Road, Luoyang, 471009 Henan Province China; 3 Department of Gastroenterology, Suzhou Municipal Hospital Affiliated to Nanjing Medical University, Suzhou, 215008 China; 4 Department of Gastroenterology, The Shanghai Tenth People’s Hospital Chongming Branch, No. 66 Xiangyang East Road, Chongming, 202157 China

**Keywords:** Inflammatory bowel disease, Disease activity, Immunoglobulin A, Immunoglobulin G

## Abstract

**Background:**

Immunoglobulin A (IgA) and IgG are major components in human intestinal mucosal surface and sera, and IgA- or IgG-coated bacteria play a vital role in the intestinal homeostasis. However, the correlation of IgA, IgG and their coated bacteria with the clinical characteristics of inflammatory bowel disease (IBD) has not been fully clarified.

**Methods:**

The levels of soluble IgA and IgG in sera and feces were detected by ELISA, and the percentage of IgA- and IgG-coated bacteria in feces was analyzed by flow cytometry. Crohn’s disease activity index (CDAI) and Simple Endoscopic Score for Crohn’s disease (SES-CD) for Crohn’s disease (CD) or Mayo score and ulcerative colitis endoscopic index of severity (UCEIS) for ulcerative colitis (UC), erythrocyte sedimentation rate (ESR) and C-reactive protein (CRP) were used to evaluate the disease activity.

**Results:**

178 patients with CD, 75 patients with UC and 41 healthy donors were recruited in this study. We found that the concentrations of soluble IgA and IgG in feces of active IBD patients were significantly higher than those in healthy controls and that the levels of soluble IgA and IgG in feces from IBD patients were positively correlated with CRP, ESR, Mayo score, UCEIS, SES-CD, and CDAI, respectively. Moreover, we also observed that the percentage of IgA- and IgG-coated bacteria markedly increased in feces of IBD patients, especially in CD patients at the age of 17 to 40 years old, with terminal ileal lesions and perianal lesions, as well as from E2 UC patients, and was closely associated with disease activities.

**Conclusions:**

The levels of soluble IgA and IgG and the percentage of IgA- and IgG-coated bacteria strikingly increase in feces of IBD patients and correlate with disease activity.

## Background

Inflammatory bowel diseases (IBD) are chronic remittent inflammatory conditions in gastrointestinal tract, including ulcerative colitis (UC) and Crohn’s disease (CD) [1, 2]. It is believed that intestinal infections, epithelial barrier disruption, dysregulated mucosal immune response to commensal microbiota and genetic variation are involved in the pathogenesis of IBD [3, 4]. Importantly, both innate immune response and adaptive immune response are found to play a vital role in the development of intestinal mucosal inflammatory damage [5–7]. However, the golden standard for the diagnosis of UC or CD is still not defined, which is routinely determined by a combination of clinical, radiological, endoscopic, histological, and biochemical investigations [8, 9]. Currently, the disease surveillance includes CRP, ESR, CDAI, SES-CD, UCEIS, and Mayo score. The rise of CRP or ESR in blood refers to acute inflammation, while CDAI and Mayo scores relies on clinical evaluation by physicians. Additionally, SES-CD and UCEIS are entirely utilized to evaluate endoscopic severity of CD and UC, respectively [10, 11]. Although several invasive methods such as blood biochemical tests and endoscopic examination have been used in the clinic, novel non-invasive disease activity-monitoring techniques are needed to replace the existing techniques.

Immunoglobulin A (IgA) has been found to be the main antibody produced within the human intestines, while IgG is the most abundant antibody in peripheral blood. They both act as the major components of humoral and mucosal immunity. Mature B lymphocytes express IgM on the surface and undergo class switch recombination (CSR) and somatic hypermutation (SHM), which are activated by antigen stimulation in lymphoid follicles [12]. Both CSR and SHM are regulated by activation-induced cytidine deaminase (AID), and CSR changes the heavy chain of immunoglobulin constant region, causing the conversion from IgM into IgA, IgG or IgE, without varying antigen specificity. Moreover, SHM generates point mutation in both heavy and light chains of the variable region and produces high affinity antibodies [12, 13].

During the innate and adaptive humoral responses, masses of commensal bacteria induce T-independent IgA response, which is primarily produced from the B1b lineage [14]. Recent work has shown that IgG antibody identifies the commensal bacteria and that maternal IgG and IgA limit T helper (Th) cell immune response to mucosal commensal antigens [15]. Moreover, IgA-coated non-invasion bacteria enter into Peyer’s patches and produce a positive feedback loop of IgA induction [16]. Gut microbiota produces homeostatic IgG antibody, and this commensal-specific IgG identifies antigens and protects against infection by aiming at conserving antigen at pathogens [17]. Otherwise, microbiota-specific IgA and IgG are found to be deficient and also involved in the pathogenesis of HIV-1-associated infection, and chronic inflammation was caused by enhancing the translocation of microbial antigens from intestinal mucosal barrier into systemic circulation owing to the dwindling of IgA and IgG [18].

Previous studies have demonstrated that IgA- or IgG-coated bacteria are increased in the intestinal lumen of IBD patients [19–21] and that colonization of IgA-coated bacteria in germ-free mice has been more prone to the occurrence of experimental colitis [21, 22], thus IgA and IgG may play vital roles in the pathogenesis of IBD. However, the underlying mechanism is still unclear, and the relationship between the levels of IgA or IgG in feces and the clinical characteristics has not been clarified.

In this report, we demonstrated that the levels of soluble IgA and IgG and the percentage of IgA- and IgG-coated bacteria significantly increased in the feces of active IBD patients compared with healthy controls. More importantly, the levels of fecal soluble IgA, IgG and the percentage of IgA- and IgG-coated bacteria were closely associated with the disease activity of IBD patients. This study thus highlights the clinical implications of fecal IgA and IgG in the pathogenesis of IBD, which may be helpful in assessing and monitoring the disease activity using such a non-invasive technique.

## Methods

### Patients and volunteers

Fecal and blood samples were collected from patients with CD (115 males and 63 females, age  33.7± 12.2 years) and UC (43 males and 32 females, age 41.2 ± 14.9 years), and healthy donors (18 males and 23 females, age 33.6 ± 10.3 years) from the Department of Gastroenterology, the Shanghai Tenth People’s Hospital of Tongji University from August 2016 to October 2018. IBD was diagnosed by the conventional clinical, radiological and endoscopic features, and eventually confirmed by histological examination of ileal and colonic biopsies. All subjects met the criteria for not receiving antibiotic and probiotic therapy for at least 3 months. Characteristics of all patients are represented in Table 1. Clinical disease activity in CD patients was measured by Simple Endoscopic Score for Crohn’s disease values (SES-CD) and Crohn’s disease activity index (CDAI) for CD. UC patients were evaluated for clinical disease activity by Mayo scoring system and ulcerative colitis endoscopic index of severity (UCEIS) [8, 23]. Remission CD patients were ruled as CDAI < 150 points and SES-CD ≤ 2 points, and remission UC patients were defined as Mayo score ≤ 2 points and UCEIS ≤ 1 point [10]. CD and UC patients were categorized according to the Montreal classification system [24]. Patients with CD were classified as A1 (≤ 16 years), A2 (17–40 years), and A3 (> 40 years) by age. The lesions were divided into L1 (ileal disease), L2 (colonic disease), and L3 (ileocolonic disease), and the disease behaviors were segmented into B1 (non-structuring and non-penetrating), B2 (structuring), B3 (penetrating), and P (perianal disease). Patients with UC were classified as E1 (proctitis), E2 (left-sided colitis), and E3 (extensive colitis).

**Table 1:** Characteristics of the patients with IBD

	HC	CD	UC
Number of patients	41	178	75
Age (years)	33.6 ± 10.3	33.7 ± 12.2	41.2 ± 14.9
Gender			
Male	18	115	43
Female	23	63	32
Active/remission		80/98	54/21
Disease duration (months)		35.3 ± 16.3	34.4 ± 18.1
Current therapy			
5-Aminosalicylates		63	49
Salazosulfapyridine		1	3
Azathioprine		47	6
Glucocorticoids		18	20
Methotrexate		6	4
Biologics		63	2
Nutritional therapy		45	6
Age (CD)^a^			
A1		14	
A2		122	
A3		42	
Disease location (CD)^a^			
L1		57	
L2		49	
L3		72	
Disease behavior (CD)^a^			
B1		99	
B2		65	
B3		14	
P		33	
Disease phenotype (UC)^a^			
E1			16
E2			23
E3			34

^a^According to the Montreal classification system

### Reagents

The following antibodies used for ELISA including goat anti-human IgA and IgG, and biotinylated anti-IgA and IgG were purchased from KPL (Gaithersburg, MD, USA). TMB and HRP were purchased from BioLegend (San Diego, CA, USA). The following reagents were used for flow cytometry including FITC-conjugated anti-human IgA (clone IS11-8E10, Miltenyi Biotec; Gladbach, Germany), PE-conjugated anti-human IgG (clone IS11-3B2.2.3, Miltenyi Biotec), and Live/dead dye (Life Technologies; Carlsbad, CA, USA).

### Measurement of soluble IgA and IgG in feces and sera by ELISA

Fecal and serum IgA and IgG were determined using the method as described previously [25]. Fecal samples were weighed and homogenized in PBS containing 20 mM EDTA, 0.04 mg/ml soybean trypsin inhibitor, and 2 mM PMSF. The supernatants were collected and stored at − 80 °C after centrifugation. ELISA was performed according to a protocol described previously [25]. Briefly, ELISA plates were coated with goat anti-human IgA or goat anti-human IgG (diluted at 1:10,000) and washed with an ELISA plate washer. Plates were then blocked and washed, and the samples for measurement of IgA and IgG from healthy controls, IgG from IBD patients, and IgA from IBD patients were diluted with 1% BSA in 1:1000, 1:1200, and 1:3200, respectively. Experimental samples and standards were added to the wells and incubated at room temperature for 2 h, and biotinylated anti-IgA or IgG (diluted in 1:16,000) with HRP-conjugated streptavidin was added for 1 h. Finally, the plates were developed in the dark using TMB substrate and analyzed by an ELISA plate reader according to the manufacturer’s instructions.

### Analysis of fecal IgA- and IgG-coated bacteria by flow cytometry

Fecal IgA- and IgG-coated bacteria were analyzed using the method as described previously [21, 25]. Briefly, 100 mg feces were homogenized in 1 ml PBS and centrifuged to separate large particles from bacteria. The supernatants were collected and washed with 1 ml PBS containing 1% bovine serum albumin. After an additional wash, bacterial pellets were incubated with blocking buffer. Bacteria were then stained with FITC-conjugated anti-human IgA, PE-conjugated anti-human IgG and Live/dead dye for 30 min on ice. Flow cytometry was performed on FACS Canto™ II and analyzed by FlowJo software according to the manufacturer’s instructions (Becton–Dickinson; CA, USA). FSC and SSC were set to logical scaling, and samples were first gated as FSC^+^ SSC^+^ Live/dead dye^−^ population and then gated FITC^+^ and PE^+^ population for IgA- and IgG-coated bacteria, respectively.

### Statistical analysis

All data were expressed as absolute and/or relative frequencies, and mean ± SD was presented on graphs. One-way ANOVA in Prism 6.0 (GraphPad, San Diego, CA) was used to determine levels of significance for comparisons between groups. The correlation was analyzed by Spearman’s correlation analysis. *P*-values of < 0.05 were considered to be statistically significant.

## Results

### The levels of fecal soluble IgA and IgG increase in active IBD patients

**Fig. 1:**
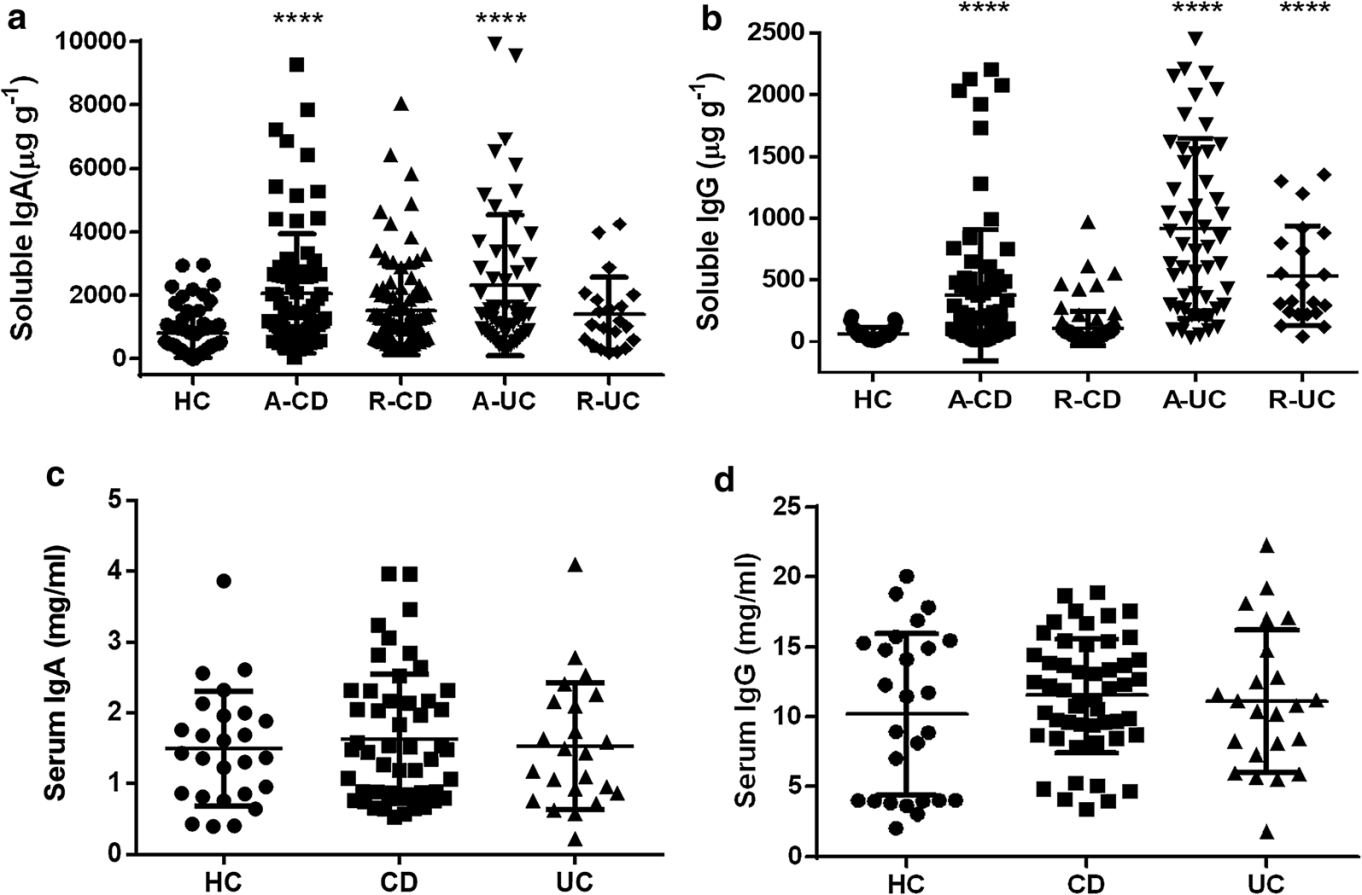
The levels of soluble IgA and IgG in feces and sera from IBD patients. Serum and fecal pellets were collected from healthy controls (HC, n = 41), active CD (A-CD, n = 80), CD in remission (R-CD, n = 98), active UC (A-UC, n = 43), and UC in remission (R-UC, n = 32), and the levels of IgA and IgG were measured by ELISA. Fecal IgA and IgG production from HC and patients with CD and UC (**a**, **b**). Serum IgA and IgG production from HC and IBD patients (**c**, **d**). *P < 0.05, ***P < 0.001, ****P < 0.0001

We first collected serum and fecal samples from IBD patients and healthy donors, and analyzed the levels of IgA and IgG by ELISA. As shown in Fig. 1, the levels of soluble IgA and IgG significantly increased in feces of active CD and UC patients compared with healthy controls (Fig. 1a, b). However, there was no statistical difference in the levels of serum IgA and IgG between IBD patients and healthy controls (Fig. 1c, d). These data indicate that soluble IgA and IgG are conspicuously up-regulated in feces of active IBD patients.

### Fecal soluble IgA and IgG are positively correlated with the disease activity of CD

**Fig. 2:**
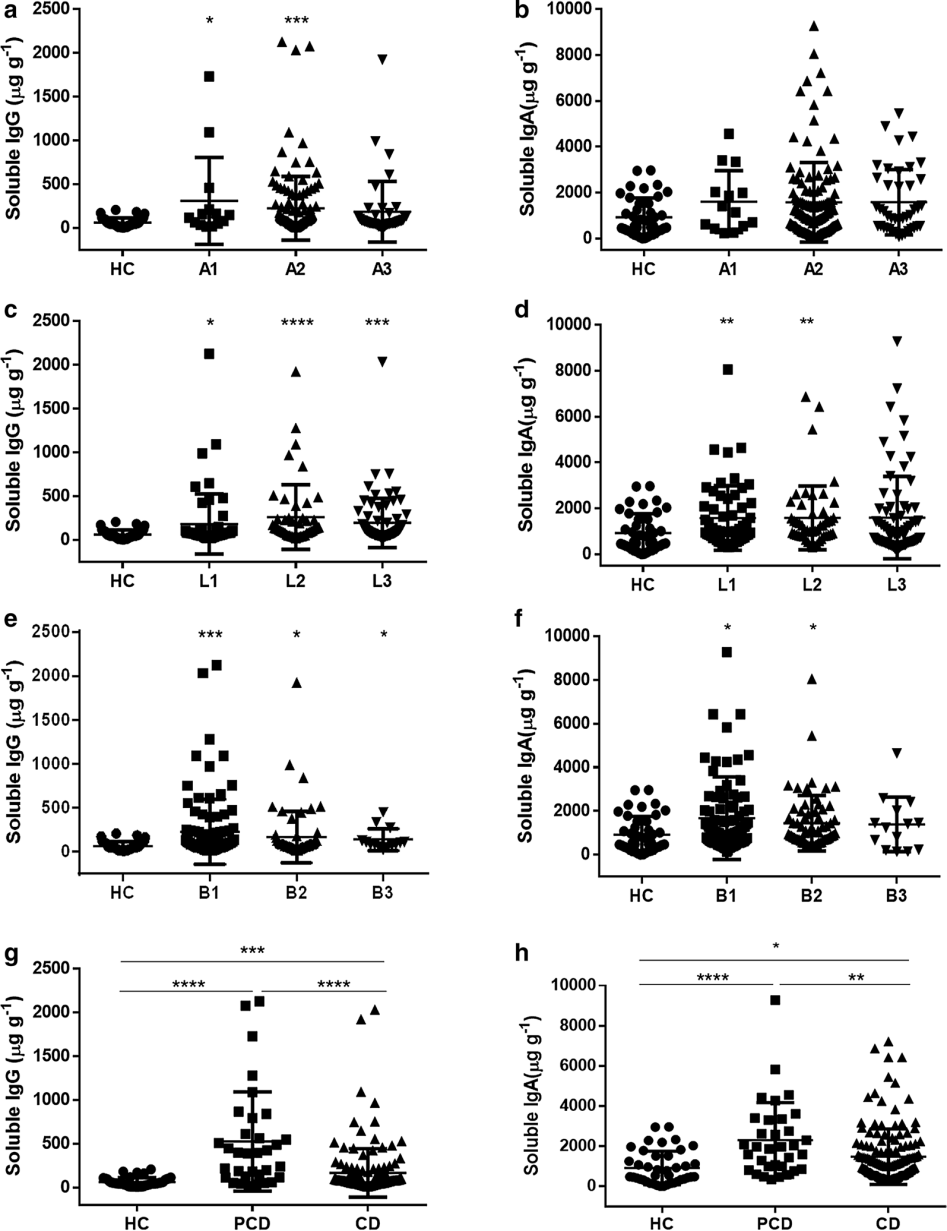
The levels of soluble IgA and IgG in feces of active CD patients according to the Montreal classification. The levels of soluble IgA and IgG in feces of active CD patients were measured by ELISA, and clinical relevance to age, disease location, disease behavior, and perianal lesions was then evaluated, respectively. Soluble IgA and IgG production in feces of HC (n = 41), A1 CD (n = 14), A2 CD (n = 122), and A3 CD (n = 42) (**a**, **b**). Soluble IgA and IgG production in feces of HC (n = 41), L1 CD (n = 57), L2 CD (n = 49), and L3 CD (n = 72) (**c**, **d**). Soluble IgA and IgG production in feces of HC (n = 41), B1 CD (n = 99), B2 CD (n = 65), and B3 CD (n = 14) (**e**, **f**). Soluble IgA and IgG production in feces of HC (n = 41), PCD (n = 33), and CD (n = 145) (**g**, **h**). *P < 0.05, **P < 0.01, ***P < 0.001, ****P < 0.0001

**Fig. 3:**
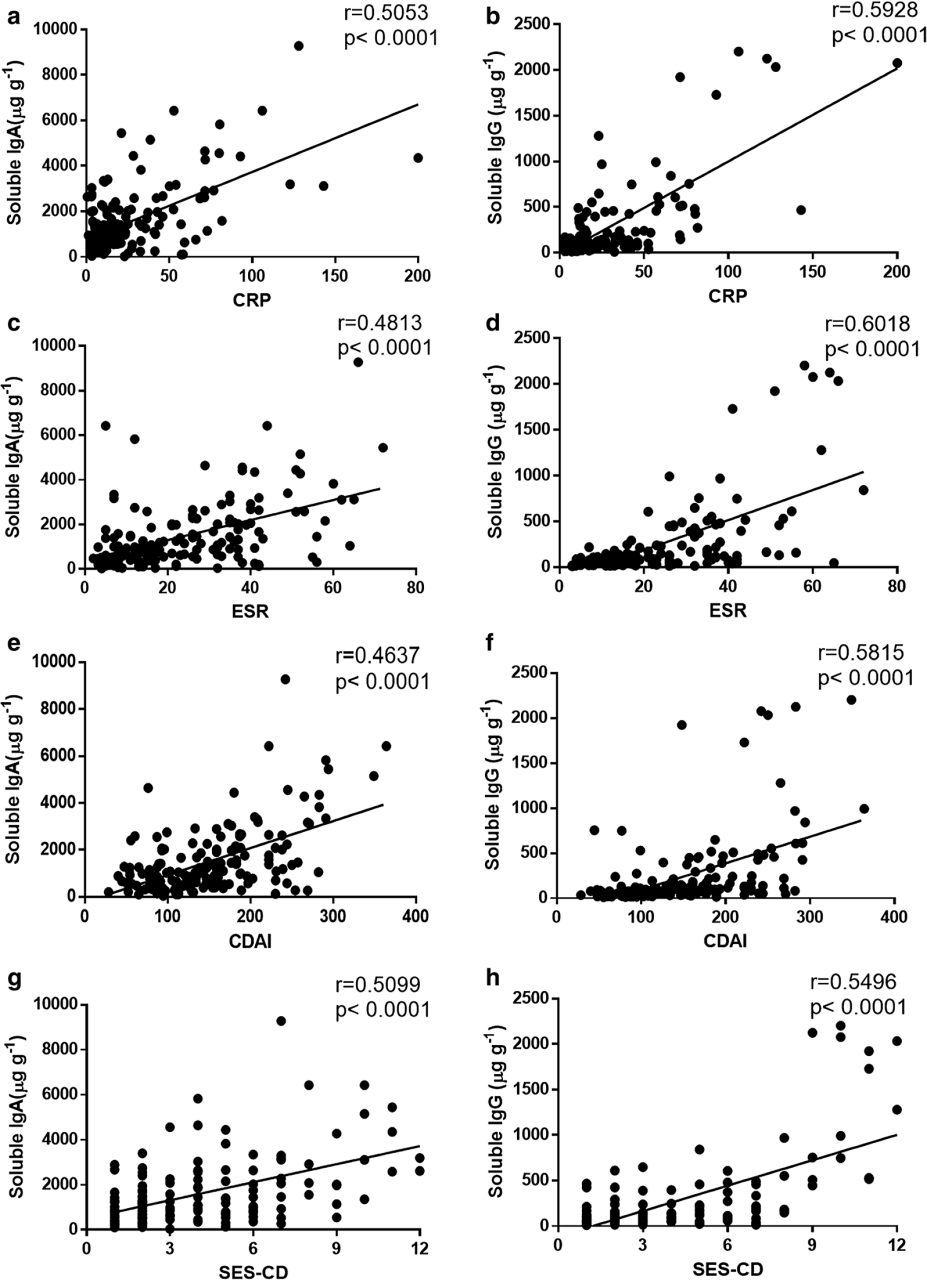
Clinical significance of soluble IgA and IgG to the disease activity, CRP and ESR in CD patients. **a**, **b** Correlations between CRP and fecal IgA (**a**) or IgG (**b**) levels in CD patients (n = 163), respectively. **c**, **d** Correlations between ESR and fecal IgA (**c**) or IgG (**d**) levels in CD patients (n = 165). **e**–**h** Correlations between CDAI (**e**, **f**) or SES-CD (**g**, **h**) and fecal IgA or IgG levels in CD patients (n = 171). Spearman’s correlation analysis was performed. *P* value is shown in each panel

To further investigate the clinical implication of the concentrations of fecal IgA and IgG, we analyzed the disease behaviors in CD patients according to the Montreal classification. Our data showed that the levels of soluble IgG were raised in A1 (≤ 16 years), A2 (17–40 years), ileal disease (L1), colonic disease (L2), ileocolonic disease (L3), non-structuring and non-penetrating (B1), structuring (B2), penetrating (B3), and perianal disease (PCD) patients (Fig. 2a, c, e and g). Besides, soluble IgA was observed to be increased in L1, L2, B1, B2 and PCD patients compared with healthy controls or non-perianal CD patients (Fig. 2b, d, f and h). CDAI, SES-CD, ESR and CRP are routinely used to evaluate the disease activity of CD patients. As shown in Fig. 3, the levels of soluble IgA or IgG were positively correlated with CRP and ESR, respectively (r = 0.5053, *P* < 0.0001 and r = 0.4813, *P* < 0.0001 for soluble IgA; r = 0.5928, *P* < 0.0001 and r = 0.6018, *P* < 0.0001 for soluble IgG, respectively) (Fig. 3a–d). Moreover, we found that the levels of soluble IgA and IgG were associated with CDAI (r = 0.4637, *P* < 0.0001 for soluble IgA; r = 0.5815, *P* < 0.0001 for soluble IgG, respectively) (Fig. 3e, f). SES-CD was also positively correlated with the levels of soluble IgA and IgG (r = 0.5099, *P* < 0.0001 for soluble IgA; r = 0.5496, *P* < 0.0001 for soluble IgG, respectively) (Fig. 3g, h). Therefore, these data suggest that the concentrations of fecal IgA and IgG are closely associated with the disease behaviors and the disease severity of CD patients.

### The levels of fecal soluble IgA and IgG are associated with the disease activity in patients with UC

**Fig. 4:**
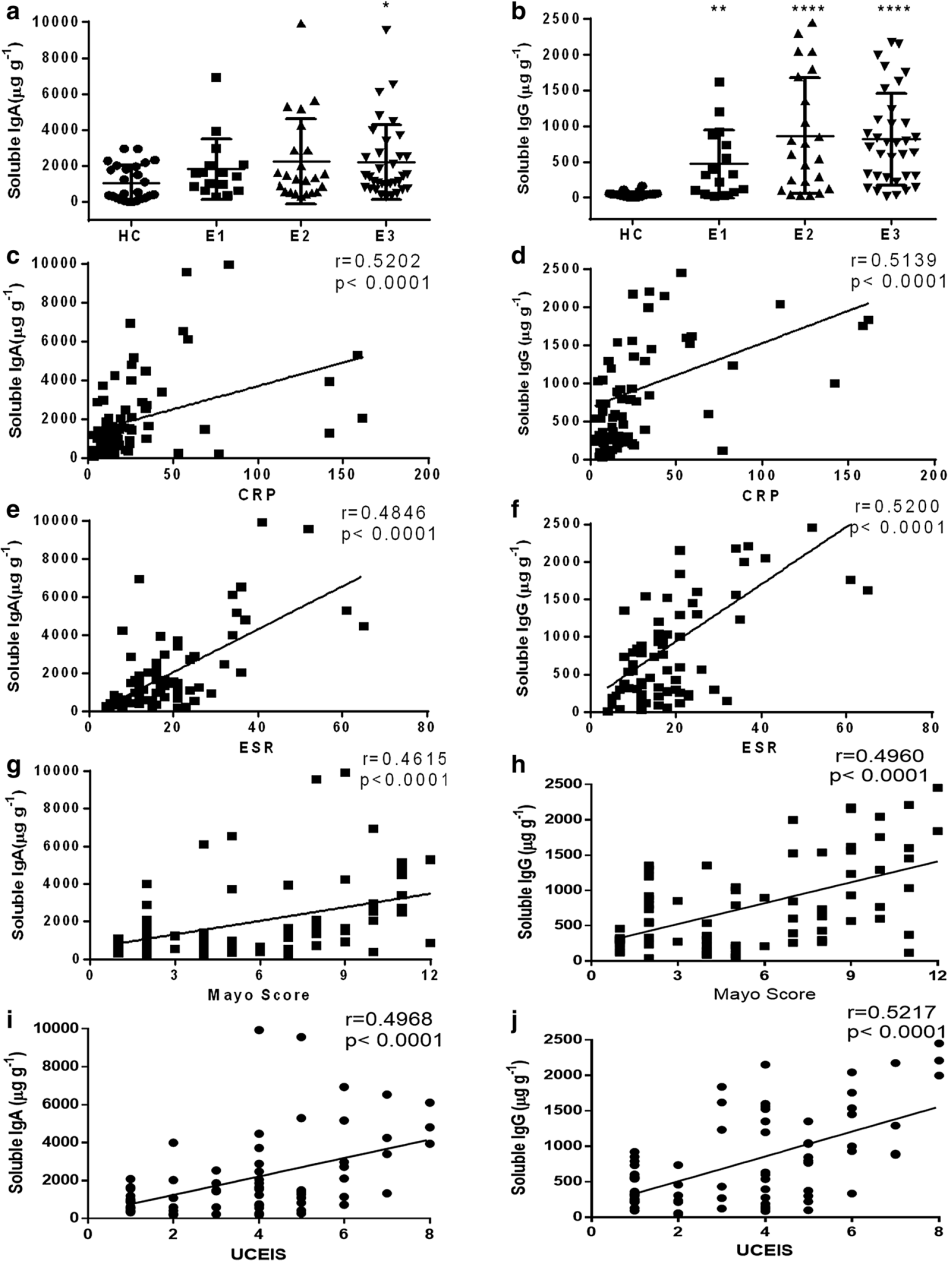
The levels of soluble IgA and IgG in feces from UC patients. **a**, **b** The levels of IgA (**a**) and IgG (**b**) were evaluated in feces of HC (n = 41), E1 UC (n = 16), E2 UC (n = 23), and E3 UC (n = 34) patients according to disease behaviors. **c**, **d** Correlations between CRP and fecal IgA (**c**) or IgG (**d**) levels in UC patients (n = 75), respectively. **e**, **f** Correlations between ESR and fecal IgA (**e**) or IgG (**f**) levels in UC (n = 75). **g**–**j** Correlations between Mayo score (**g**, **h**) or UCEIS (**i**, **j**) and fecal IgA or IgG levels in UC patients (n = 73). Spearman’s correlation analysis was performed. *P* value is shown in each panel. *P < 0.05, ****P < 0.0001

We then analyzed the association of fecal IgA and IgG with the disease behaviors in UC patients. Figure 4a, b shows that the levels of soluble IgA were significantly increased in extensive colitis patients (E3), while soluble IgG was notably enhanced in all UC patients. Soluble IgA and IgG were observed to be positively associated with CRP and ESR, respectively (r = 0.5202, *P* < 0.0001, r = 0.4846, *P* < 0.0001 for soluble IgA; r = 0.5139, *P* < 0.0001 and r = 0.52, *P* < 0.0001 for soluble IgG, respectively) (Fig. 4c–f). Mayo scoring system and UCEIS are routinely used for assessment of the disease activity of UC patients. Figure 4g–j illustrates a positive correlation between soluble IgA or IgG and Mayo score or UCESI (r = 0.4615, *P *< 0.0001 for soluble IgA; r = 0.496, *P* < 0.0001 for soluble IgG in Mayo score; r = 0.4968, *P* < 0.0001 for soluble IgA; r = 0.5217, *P* < 0.0001 for soluble IgG in UCEIS). These data demonstrate that the concentrations of fecal IgA and IgG are also related to the disease behaviors and the disease severity of UC patients.

### The percentage of IgA- and IgG-coated bacteria in feces markedly enhances in active IBD patients

**Fig. 5:**
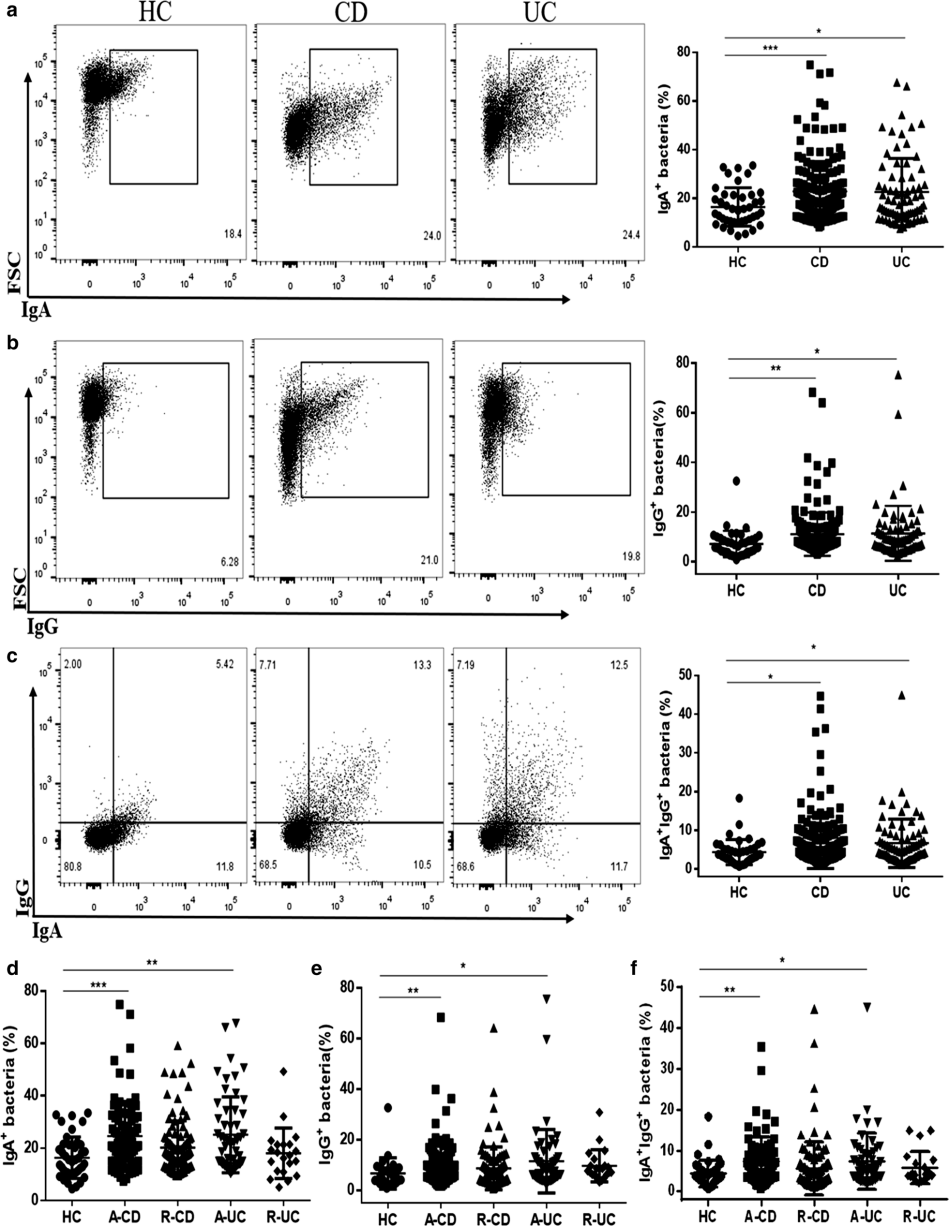
The percentage of IgA- and IgG-coated bacteria in feces of IBD patients. IgA-, IgG- and IgA/IgG-coated bacteria were determined in feces of HC and active IBD patients by flow cytometry. **a**–**c** Fluorescence-activated cell sorting (FACS) profiles of IgA-, IgG- and IgA/IgG-coated bacteria from HC (n = 41), active CD (n = 80) and active UC patients (n = 54), respectively. **d**–**f** The percentage of IgA-, IgG- and IgA/IgG-coated bacteria in feces of HC (n = 41), A-CD (n = 80), R-CD (n = 98), A-UC (n = 54) and R-UC (n = 21) patients, respectively. *P < 0.05, **P < 0.01, ***P < 0.001

The percentage of IgA- and IgG-coated bacteria was determined by flow cytometry. As shown in Fig. 5a–c, the percentage of IgA- and IgG-coated bacteria greatly increased in CD and UC patients than that in healthy controls. We found that fecal microbiota contained 22.86 ± 12.23% of IgA-coated bacteria, 11.13 ± 8.74% IgG-coated bacteria, and 6.81 ± 6.72% of IgA/IgG-coated bacteria, respectively, in CD patients, and 22.53 ± 13.83% of IgA-coated bacteria, 11.43 ± 11.07% of IgG-coated bacteria, and 6.68 ± 6.27% of IgA/IgG-coated bacteria, respectively, in UC patients. For further analysis, we found that the percentage of IgA- and IgG-coated fecal bacteria was higher in active CD and UC patients than that in healthy controls (Fig. 5d–f). Thus, these data illustrate that the activity of disease is probably related to the percentage of IgA and IgG-coated bacteria in feces of IBD patients.

### The percentage of IgA- and IgG-coated bacteria is positively correlated with the activity of CD

**Fig. 6:**
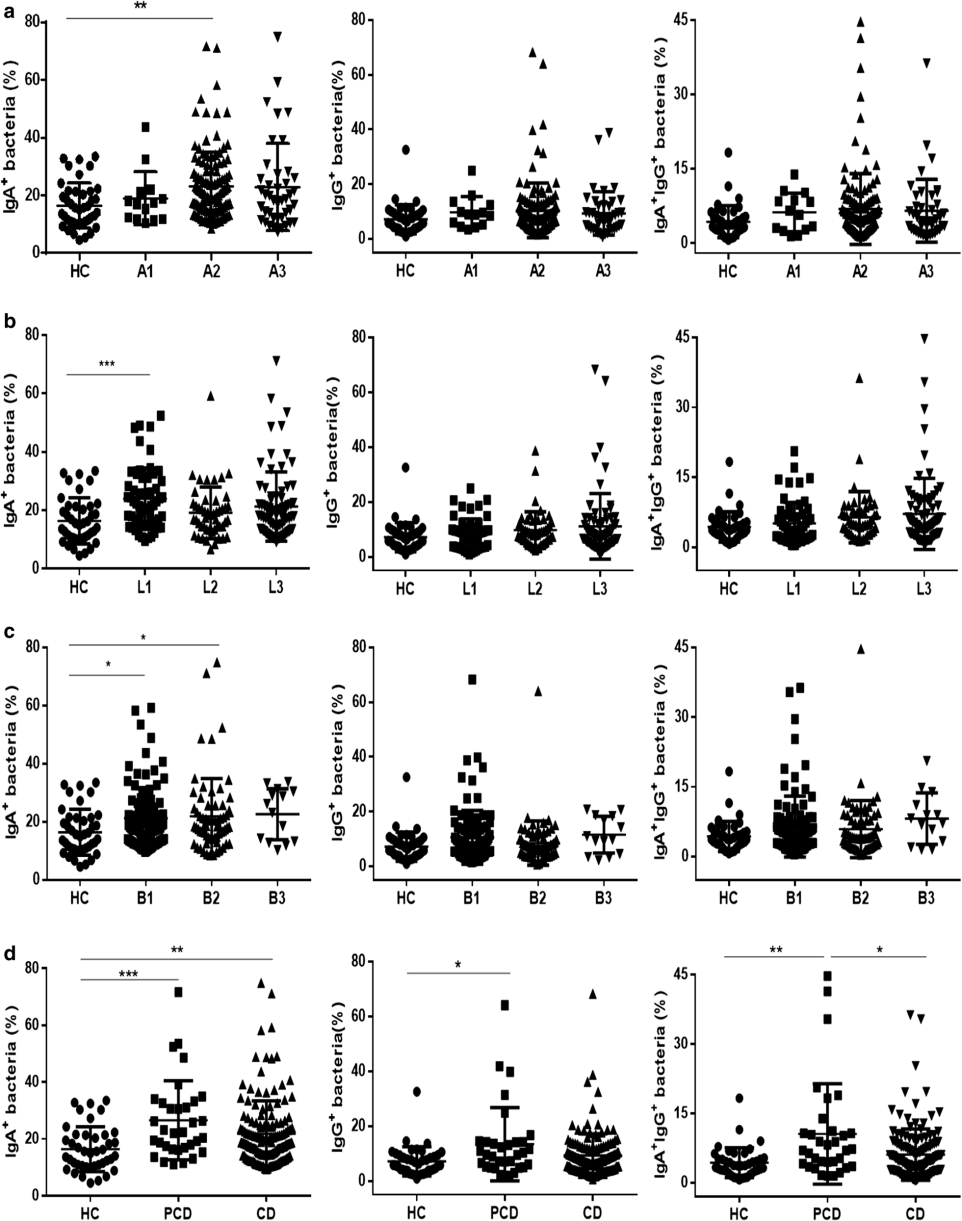
Clinical significance of the percentage of IgA- and IgG-coated bacteria in feces of CD patients. IgA-, IgG-coated bacteria and IgA/IgG-coated bacteria were determined by flow cytometry, and clinical relevance of these bacteria was evaluated in respect to age, location, disease behavior and perianal lesions, respectively. **a** The percentage of IgA-, IgG- and IgA/IgG-coated bacteria in feces of HC (n = 41), A1 CD (n = 14), A2 CD (n = 122), and A3 CD (n = 42) patients. **b** The percentage of IgA-, IgG- and IgA/IgG-coated bacteria in feces of HC (n = 41), L1 CD (n = 57), L2 CD (n = 49), and L3 CD (n = 72) patients. **c** The percentage of IgA-, IgG- and IgA/IgG-coated bacteria in feces of HC (n = 41), B1 CD (n = 99), B2 CD (n = 65), and B3 CD (n = 14) patients. **d** The percentage of IgA-, IgG- and IgA/IgG-coated bacteria in feces of HC (n = 41), PCD (n = 33), and CD (n = 145). *P < 0.05, **P < 0.01

According to Montreal classification, we analyzed the associations of percentage of IgA- and IgG-coated bacteria in feces of CD patients with age, location, and disease behavior, respectively. Interestingly, we found that the percentage of IgA-coated bacteria dramatically increased in CD patients at the age of 17 to 40 years old (A2, Fig. 6a), with ileal lesions (L1, Fig. 6b), non-structuring and non-penetrating (B1), and structuring (B2, Fig. 6c) compared to other disease subtypes, while no statistical differences were found in percentage of IgG-coated bacteria and IgA/IgG-coated bacteria in feces of CD patients between different subgroups (Fig. 6a–c). Moreover, we also observed that the percentage of IgA-, IgG- and IgA/IgG-coated bacteria was up-regulated in perianal CD (PCD) patients compared with that in non-perianal CD patients and healthy controls (Fig. 6d). However, the percentage of IgG- and IgA/IgG-coated bacteria has no difference among the disease behaviors with or without structuring and penetration (Fig. 6c).

**Fig. 7:**
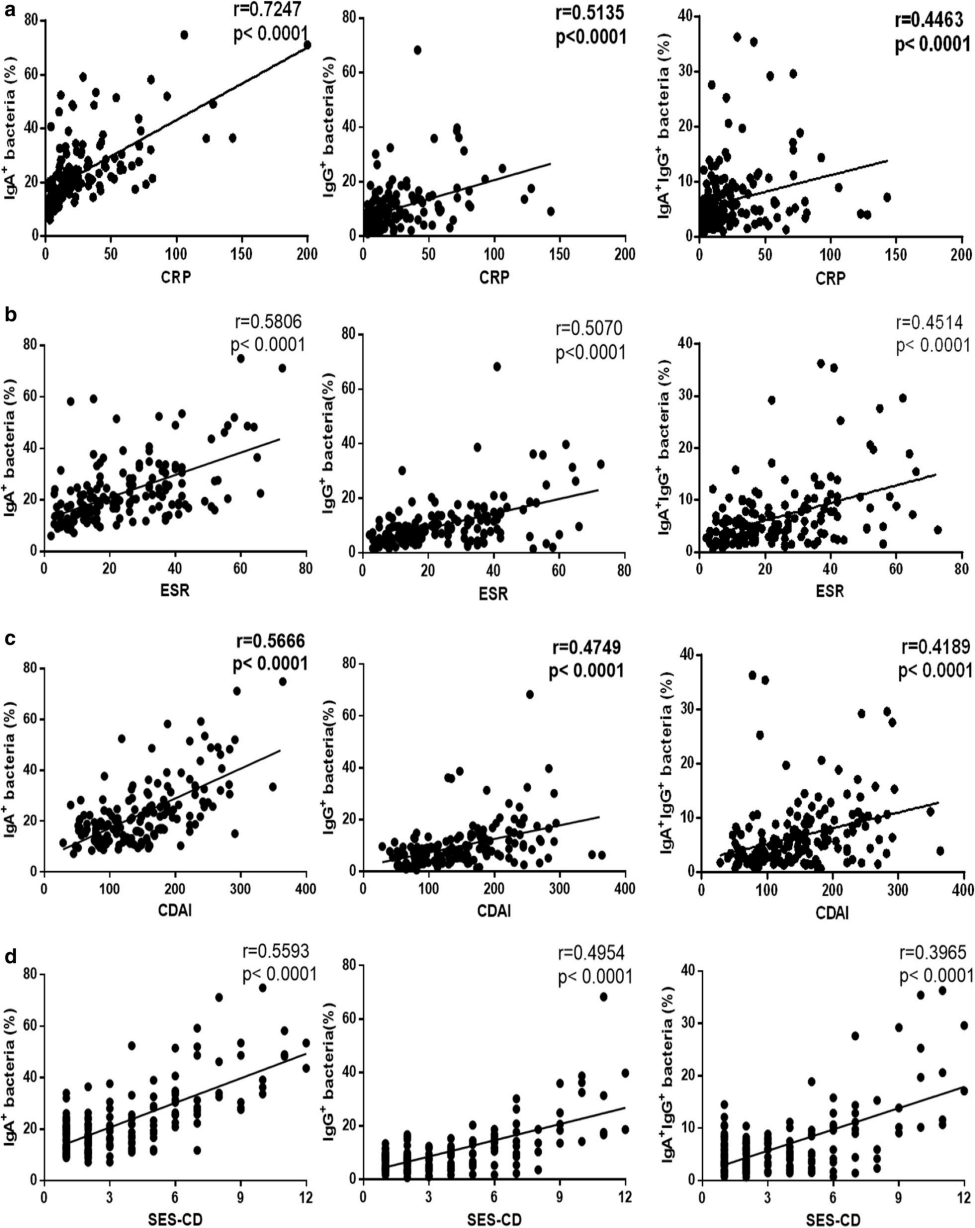
The relationship between the levels of fecal IgA- or IgG-coated bacteria and CRP, ESR and the disease activity, respectively, in CD patients. **a** Correlations between CRP and the percentage of fecal IgA-, IgG- and IgA/IgG-coated bacteria in CD patients (n = 163). **b** Correlations between ESR and the percentage of fecal IgA-, IgG- and IgA/IgG-coated bacteria in CD patients (n = 165). **c** Correlations between CDAI and the percentage of fecal IgA-, IgG- and IgA/IgG-coated bacteria in CD patients (n = 171). **d** Correlations between SES-CD and the percentage of fecal IgA-, IgG- and IgA/IgG-coated bacteria in CD patients (n = 171). Spearman’s correlation analysis was performed. P value is shown in each panel

We next analyzed the relationship between the percentage of IgA- and IgG-coated bacteria and the disease activity of CD patients. As shown in Fig. 7a, b, the percentage of IgA- and IgG-coated bacteria appeared to be notably positive correlated with CRP and ESR, respectively (r = 0.7247, *P* < 0.0001 and r = 0.5806, *P* < 0.0001 for IgA-coated bacteria; r = 0.5135, *P* < 0.0001 and r = 0.5070, *P* < 0.0001 for IgG-coated bacteria, respectively). A weak correlation was also present between percentage of IgA/IgG-coated bacteria and CRP or ESR (r = 0.4463, *P* < 0.0001 and r = 0.4514, *P* < 0.0001, respectively). Moreover, a positive association was detected between CDAI and the percentage of IgA- and IgG-coated bacteria in CD patients (r = 0.5666, *P* < 0.0001 for IgA-coated bacteria; r = 0.4749, *P* < 0.0001 for IgG-coated bacteria; r = 0.4189, *P* < 0.0001 for IgA/IgG-coated bacteria) (Fig. 7c). We also found that SES-CD was positively related to the percentages of IgA- and IgG-coated bacteria (r = 0.5593, *P* < 0.0001 for IgA-coated bacteria; r =  0.4954, *P* < 0.0001 for IgG-coated bacteria; r = 0.3965, *P* < 0.0001 for IgA/IgG-coated bacteria) (Fig. 7d). Taken together, these results prove that IgA- and IgG-coated fecal flora are present in CD patients and associated with the disease activity.

### The percentage of IgA- and IgG-coated bacteria in feces is related to the disease activity of UC patients

**Fig. 8:**
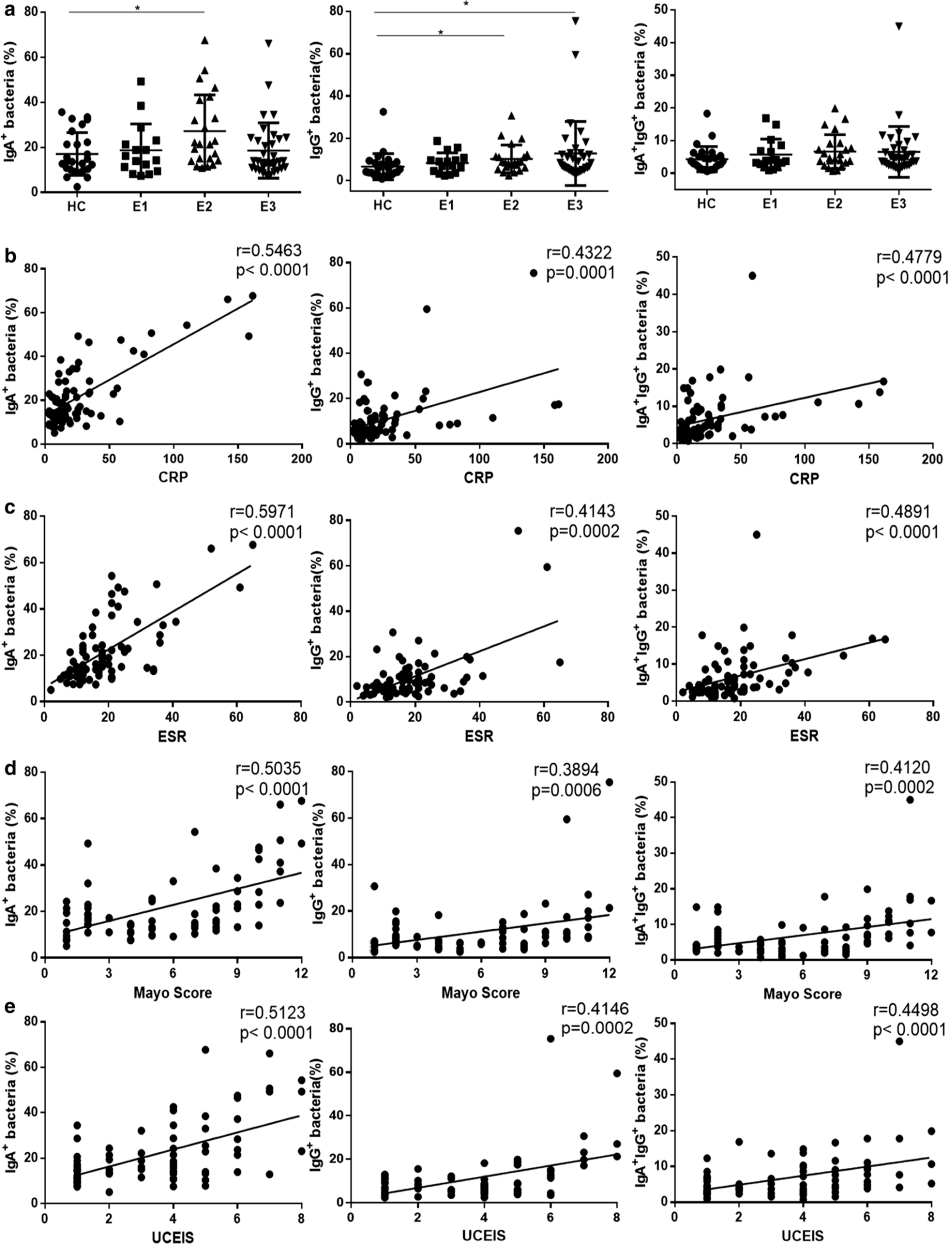
The percentages of IgA- and IgG-coated bacteria in feces of UC patients. The percentage of IgA-, IgG- and IgA/IgG-coated bacteria in feces of HC and UC patients was determined by flow cytometry. **a** The percentages of IgA-, IgG- and IgA/IgG-coated bacteria were evaluated in feces of HC (n = 41), E1 UC (n = 16), E2 UC (n = 23), and E3 UC (n = 34) patients according disease behaviors. **b** Correlations between CRP and the percentage of IgA-, IgG- and IgA/IgG-coated bacteria in feces of UC patients (n = 75). **c** Correlations between ESR and the percentage of IgA-, IgG- and IgA/IgG-coated bacteria in feces of UC patients (n = 75). **d** Correlations between Mayo score and the percentage of IgA-, IgG- and IgA/IgG-coated bacteria in feces of UC patients (n = 73). **e** Correlations between UCEIS and the percentage of IgA-, IgG- and IgA/IgG-coated bacteria in feces of UC patients (n = 73). Spearman’s correlation analysis was performed. *P* value is shown in each panel. *P < 0.05

We next studied the clinical relevance of the percentage of IgA- and IgG-coated bacteria to disease behaviors in UC patients, and found that the percentage of IgA-coated bacteria increased in patients with left-side colitis (E2), while E2 UC and patients with extensive colitis (E3) displayed higher percentage of IgG-coated bacteria compared with healthy controls (Fig. 8a). Otherwise, positive correlations were found between percentage of IgA- or IgG-coated bacteria and CRP or ESR (r = 0.5463, P < 0.0001 and r = 0.5971, *P* < 0.0001 for IgA-coated bacteria; r = 0.4322, *P* = 0.0001 and r = 0.4143, *P* = 0.0002 for IgG-coated bacteria; r = 0.4779, *P* < 0.0001 and r = 0.4891, *P* < 0.0001, for IgA/IgG-coated bacteria, respectively) (Fig. 8b, c). Additionally, we observed an association between percentage of IgA- and IgG-coated fecal bacteria with Mayo score and UCEIS in UC patients (r = 0.5035, *P* < 0.0001 and r = 0.5123, *P* < 0.0001 for IgA-coated bacteria; r = 0.3894, *P* = 0.0006 and r = 0.4146, *P* = 0.0002 for IgG-coated bacteria; r = 0.412, *P* = 0.0002 and r = 0.4498, *P* < 0.0001 for IgA/IgG-coated bacteria, respectively) (Fig. 8b–e). These data provide evidences that the percentage of IgA- and IgG-coated bacteria is elevated in UC patients and associated with the disease activity.

## Discussion

In this report, we demonstrated that the levels of soluble IgA or IgG and the percentage of IgA- or IgG-coated bacteria in feces were significantly increased in active IBD patients compared with healthy controls. The concentrations of soluble IgA and IgG in PCD patients were markedly elevated and the percentage of IgA-coated bacteria in ileal CD (L1) patients were also enhanced, consistent with previous studies [14]. Although previous reports have demonstrated that the number of IgA- or IgG-coated bacteria in IBD patients was higher than that in healthy controls [19–21], soluble IgA and IgG in feces of IBD patients have not been depicted. Importantly, we found that the levels of fecal IgA and IgG were positively correlated with CRP, ESR, CDAI, SES-CD, Mayo scores and UCEIS of patients with CD or UC, respectively, suggesting that fecal IgA and IgG may serve as an important parameter for monitoring the disease activity in IBD patients.

IgA is the dominant antibody in mammal mucosal secretion, and most IgA is secreted on the surface of the intestinal mucosa [26]. It has been reported to play a crucial role in protecting and regulating in intestinal homeostasis. The function of IgA in intestine has been thoroughly reviewed in the literatures [22, 26, 27], including immune exclusion, intercept incoming virus intracellularly, neutralization of pathogens and toxins, blocking certain bacteria adhesion or transport, preventing inflammatory damaging to the epithelial barrier. Furthermore, recent study demonstrated that microbial symbionts contribute to regulating the primary Ig repertoire in T cell-independent way, which could direct naïve B cells to augment bacterial reactive IgA production in small bowel [28]. Most intestinal IgA is induced by microbiota, and play an important role in maintaining the homeostasis of host-microbiota [27, 29, 30]. Both primitive and classical IgA are found to be involved in the protection from harmless commensal microbes and invasive commensal species [26, 29].

IgG as the main component of humoral immunity has been found in blood and extracellular fluid. It can be divided into four subclasses and enable to regulate infection of tissues. It is generally considered that IgG1 is the most abundant subclass, which can bind and neutralize toxin, activate the classical approach of the complement system, and play an important role in antibody-mediated proteolysis and antibody-dependent cell mediated cytotoxicity. In addition, the opsonization of IgG allows its recognition and ingestion by phagocytic cells, resulting in the elimination of pathogens [31, 32].

In our study, the soluble IgA and IgG were observed to be increased in feces of active IBD patients, which may be promoted by various antigenic stimuli, resulting in an increase of IgA^+^ and IgG^+^ plasma cells, followed by enhanced secretion of IgA and IgG in intestine. Besides, recent study showed that lamina propria CX3CR1^+^ macrophages, B cells, and CD8^+^ T cells coordinate IgA secretion in the small intestine, independent of retinoic acid signaling and TLR-mediated microbial recognition [33]. In general, serum immunoglobulin may potentially contribute to soluble and coated-bacteria immunoglobulin. Incubation with autologous sera has shown that serum IgG can bind to fecal bacteria in both CD patients and volunteers [20]. Therefore, theoretically, leakage of serum IgG into the intestine may result in a high proportion of IgA- and IgG-coated bacteria in bowel diseases. However, our data showed that the levels of serum immunoglobulins were normal in most IBD patients, indicating the content of serum immunoglobulins was dispensable to the increase of intestinal immunoglobulins. In the past decades, the imbalance of gut bacteria involved in the pathogenesis of IBD has been reviewed in several literatures [1–3]. Previous data have demonstrated that colonization of isolated IgA-coated bacteria in the intestines of germ-free mice tends to induce experimental colitis [21, 22]. Moreover, another study has shown that IgA-coated adherent-invasive *Escherichia coli* isolated from CD patients with spondyloarthritis can aggravate colitis or inflammatory arthritis after transplanted into interleukin-10 deficient or K/BxN mice [34]. Taken together, our studies provide evidence that changes in the composition of the intestinal flora result in an increased proportion of bacteria coated with IgA and IgG, and an enhanced humoral immune response against the intestinal antigens in active IBD patients.

Our study draws interesting findings in that the expression of fecal soluble IgA or IgG, and IgA- or IgG-coated bacteria in patients with active IBD was elevated and positively correlated with disease activity. However, this study has some limitations. Firstly, numbers of patients with IBD were on medications in the study. So, this study could not compare the levels of fecal IgA and IgG before and after treatment. Secondly, this study was conducted in a single medical center, and the number of IBD patients who met our criteria was insufficient. Further studies from several medical centers should be conducted to clarify the relationship between fecal IgA and IgG and disease activity.

## Conclusion

Our results show that the levels of fecal IgA and IgG in patients with IBD are significantly increased in comparison with healthy controls and that the amount of IgA and IgG is positively related to the degree of disease activity. The levels of soluble IgA or IgG, and IgA- or IgG-coated bacteria are closely associated with the values of CRP, ESR, CDAI, SES-CD, Mayo score, and UCEIS, respectively. Moreover, our results indicate that there are increased immunogenic bacteria in the feces of active IBD patients compared with healthy controls. Currently, the surveillance of disease activity in IBD patients is mainly dependent on endoscopy and blood biochemical tests, while some patients have difficulty in the tolerance of such examination. Therefore, our study provides a novel strategy for detecting the disease severity by a simple and non-invasive way of detecting IgA and IgG in feces.

## Abbreviations

IgA: immunoglobulin A
IgG: immunoglobulin G
IBD: inflammatory bowel disease
CD: Crohn’s disease
UC: ulcerative colitis
CDAI: Crohn’s disease activity index
SES-CD: Simple Endoscopic Score for Crohn’s disease
UCEIS: ulcerative colitis endoscopic index of severity
ESR: erythrocyte sedimentation rate
CRP: C-reactive protein
CSR: class switch recombination
SHM: somatic hypermutation
AID: activation-induced cytidine deaminase
